# Anesthetic Management in the Elective Separation of Omphalopagus Conjoined Twins With Shared Hepatic Parenchyma: A Case Report

**DOI:** 10.7759/cureus.102191

**Published:** 2026-01-24

**Authors:** Inês Godinho, Jorge Paulos, Ana Pinto Carneiro

**Affiliations:** 1 Anesthesiology, Unidade Local de Saúde de São José - Centro Hospitalar Universitário de Lisboa Central, Lisbon, PRT; 2 Anesthesiology and Reanimation, Centro Hospitalar Universitário de Lisboa Central, Lisbon, PRT; 3 Anesthesiology, Unidade Local de Saúde de São José, Lisbon, PRT

**Keywords:** case report, conjoined twins, hepatic separation, omphalopagus, pediatric anesthesia

## Abstract

Separation of conjoined twins is a rare and complex surgical endeavor with significant anesthetic implications, particularly in omphalopagus twins, given the risk of massive hemorrhage during hepatic transection and hemodynamic instability from cross-circulation. Blood management strategies include the use of tranexamic acid, viscoelastic monitoring, and preparation for rapid transfusion. We report the anesthetic management of the elective surgical separation of three-year-old female omphalopagus conjoined twins with shared hepatic parenchyma. Extensive multidisciplinary planning, simulation-based preparation, independent anesthesia teams, invasive monitoring, and proactive patient blood management strategies were implemented. Careful preoperative optimization, structured intraoperative coordination, and adherence to anesthetic principles derived from prior case-based evidence were central to maintaining hemodynamic and metabolic stability throughout the perioperative period. This report adds to the limited body of literature on elective omphalopagus separation involving hepatic division.

The key learning points are as follows: Separation of omphalopagus conjoined twins presents significant anesthetic challenges, primarily related to shared hepatic parenchyma and the risk of major hemorrhage. Detailed preoperative imaging, including hepatic angiography, is essential to define vascular anatomy and anticipate transfusion requirements. Assessment of cross-circulation is critical to guide safe anesthetic drug administration and neuromuscular blockade strategies. Multidisciplinary planning with simulation-based rehearsal and strict duplication of anesthesia teams and equipment enhances perioperative safety. Proactive blood management, including viscoelastic-guided transfusion and antifibrinolytic therapy, is central to maintaining hemodynamic stability. Structured postoperative intensive care monitoring is vital for early detection and management of hepatic, renal, and infectious complications.

## Introduction

Surgical separation of omphalopagus conjoined twins represents a rare and highly complex procedure, with anesthetic management playing a central role in determining perioperative outcomes. Omphalopagus twins are typically fused at the anterior abdominal wall and frequently share hepatic parenchyma and vascular structures, which makes separation surgery particularly challenging due to the risk of massive hemorrhage, hemodynamic instability, and prolonged operative times [1,2]. Shared hepatic anatomy can lead to significant blood loss, necessitating careful transfusion planning and close monitoring for metabolic derangements such as hypothermia, acidosis, and coagulopathy.

The available evidence regarding anesthetic management in conjoined twin separation is largely derived from case reports, small case series, and narrative reviews, reflecting the condition’s rarity [3,4,5,6]. Over time, core anesthetic principles have been established, including treating each twin as an independent patient, employing two dedicated anesthesia teams, duplicating anesthesia workstations and monitoring systems, and ensuring meticulous intraoperative communication [1,3,6]. However, several aspects remain underreported or controversial, including optimal anesthetic drug titration strategies in older children, transfusion thresholds, and the practical assessment of cross-circulation in real-world settings.

Shared hepatic parenchyma represents one of the most significant anesthetic challenges in omphalopagus conjoined twins. Previous reports emphasize the importance of detailed preoperative imaging to define hepatic anatomy and vascular sharing, anticipating major blood loss during hepatic parenchymal division, preparing for massive transfusion, initiating early invasive monitoring, and maintaining strict temperature control [2,4,5,7]. Assessment of potential cross-circulation and cautious titration of anesthetic agents are also critical to avoid unintended pharmacological effects between twins [3,8,9,10]. We report the anesthetic management of the elective surgical separation of three-year-old omphalopagus conjoined twins with shared hepatic parenchyma, highlighting perioperative challenges and management strategies within the context of previously published experience.

This article was previously presented as a poster at the World Congress of Anaesthesiologists (WCA) 2024.

## Case presentation

Patient characteristics and preoperative evaluation

We describe the case of three-year-old female omphalopagus conjoined twins (DS and BS) born following limited prenatal surveillance, with the diagnosis established at approximately five months of gestation. Delivery had occurred via cesarean section under general anesthesia, without immediate neonatal complications. Both children demonstrated age-appropriate psychomotor development.

**Figure 1:**
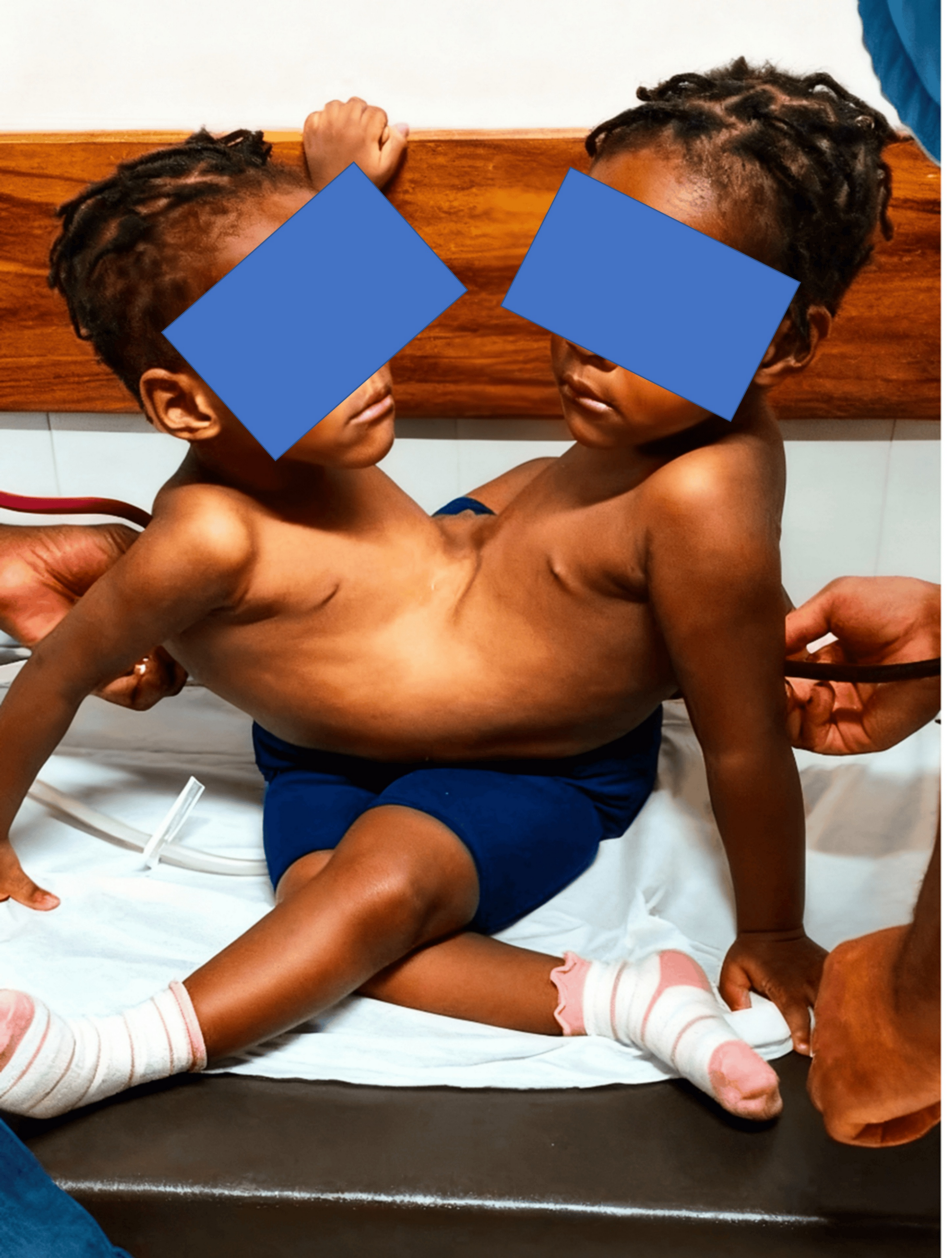
Preoperative clinical appearance, demonstrating anterior abdominal wall fusion

Anatomical evaluation revealed fusion extending from the costal margin to the perineal region (Figure 1). Cardiac and pulmonary anatomy were independent and structurally normal. The twins shared hepatic parenchyma with capsular fusion. Preoperative imaging, including contrast-enhanced CT and selective hepatic angiography, demonstrated normal individual hepatic vasculature without major vascular anomalies, and no aberrant or dominant vascular connections were identified. Pharmacologic cross-circulation testing was performed preoperatively, demonstrating minimal hemodynamic interaction between the twins during incremental doses of intravenous anesthetic agents. Additional findings included a single distal ileal segment with a shared colon, a single anorectal segment, independent kidneys with crossed ureteral ectopia, and separate bladders and genital organs. No bony fusion was identified.

Both twins had a history of recurrent urinary tract infections and iron-deficiency anemia, which were optimized before surgery in collaboration with the transfusion medicine team. The preoperative hemoglobin level was 11.6 g/dL in both patients. Cardiac evaluation showed preserved function, and both twins were classified as American Society of Anesthesiologists (ASA) physical status III because of the complexity of shared anatomy.

Multidisciplinary planning

**Figure 2:**
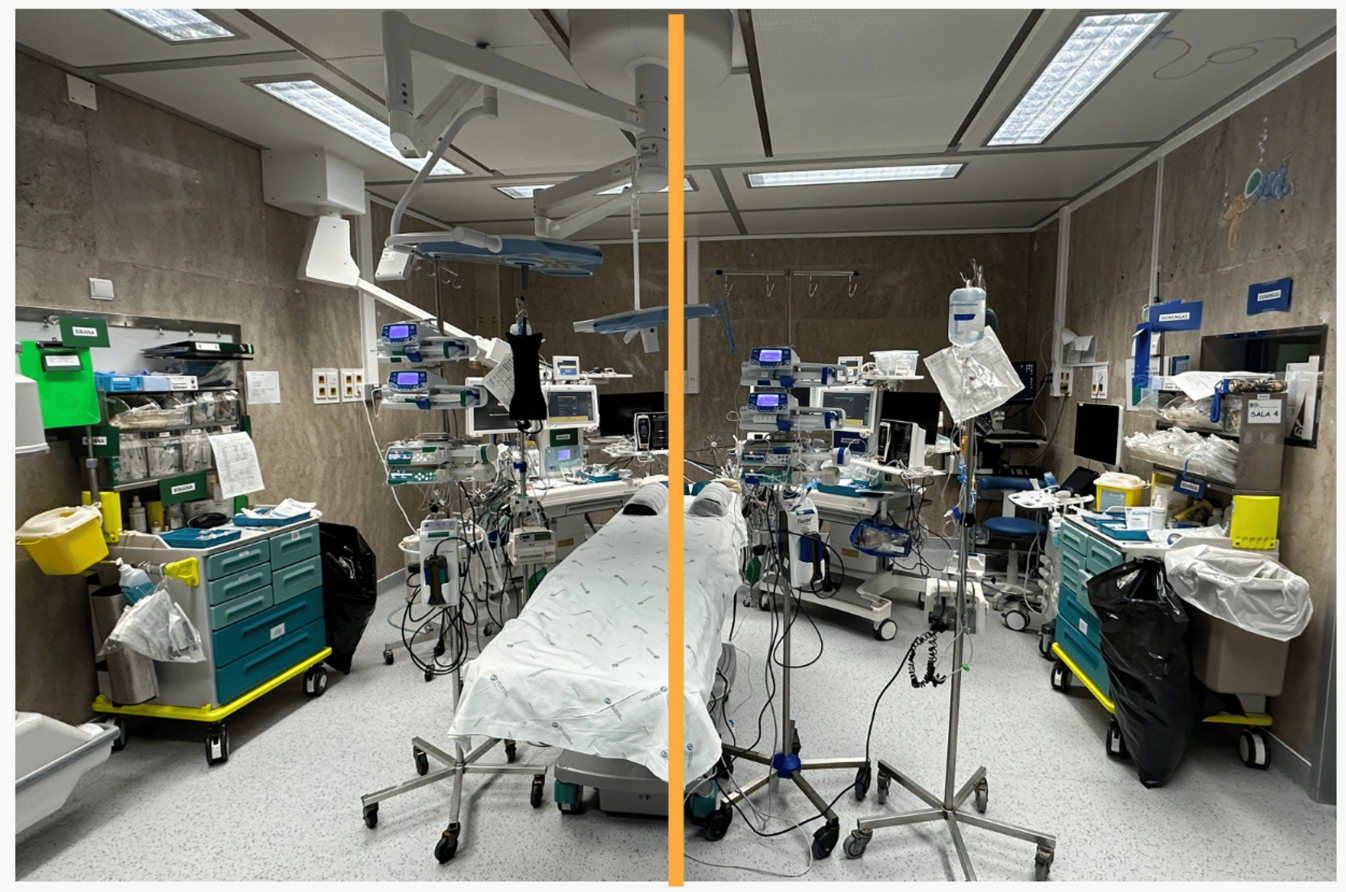
Operating room setup demonstrating complete duplication of anesthesia workstations, monitoring systems, and infusion devices, with a clear spatial division between the two anesthesia teams

Given the complexity of the planned elective separation, extensive multidisciplinary planning was undertaken [6,9]. Two independent anesthesia teams were assigned, each comprising a consultant anesthesiologist and an anesthesia trainee, with complete duplication of anesthesia workstations, monitoring equipment, medications, and infusion devices (Figure 2). Color-coded identification systems were used to prevent cross-administration of drugs, fluids, or blood products between twins [1,6,9]. Simulation-based rehearsals were conducted with predefined objectives, including management of massive hemorrhage during hepatic transection, hemodynamic instability following separation, and airway or ventilatory emergencies during repositioning.

Preoperative planning included estimation of potential blood loss based on imaging and surgical assessment, anticipating losses exceeding one circulating blood volume during hepatic division. Blood products were reserved preoperatively, including packed red blood cells, plasma, platelets, and fibrinogen [4,5,10], with predefined transfusion thresholds guided by hemoglobin levels, viscoelastic testing, and clinical hemodynamics. A pediatric-adapted massive transfusion protocol was established in advance, allowing rapid escalation of blood component therapy if required. Pediatric ICU beds were secured for both patients postoperatively.

Anesthetic management

Premedication consisted of oral midazolam (0.2 mg/kg). Standard monitoring was supplemented with invasive arterial blood pressure monitoring, which allowed prompt detection and correction of hemodynamic changes during hepatic parenchymal division, contributing to the absence of sustained hypotension or vasoactive support requirements. Bispectral index monitoring facilitated individualized titration of anesthetic depth, reducing the risk of inadvertent anesthetic overdose in the context of potential cross-circulation. Near-infrared spectroscopy enabled continuous assessment of cerebral oxygenation and supported maintenance of adequate cerebral perfusion during blood loss and patient repositioning, without evidence of cerebral desaturation.

Continuous temperature monitoring ensured strict normothermia throughout the prolonged procedure, minimizing coagulopathy and metabolic complications. Serial arterial blood gas analysis guided ventilatory management to maintain normocapnia and allowed early identification of metabolic changes, preventing acid-base derangements. Neuromuscular blockade monitoring informed cautious use of muscle relaxants, avoiding unintended prolonged paralysis in the second twin. Urinary output monitoring supported real-time assessment of intravascular volume status and renal perfusion, contributing to early recognition and management of transient postoperative renal dysfunction.

**Figure 3:**
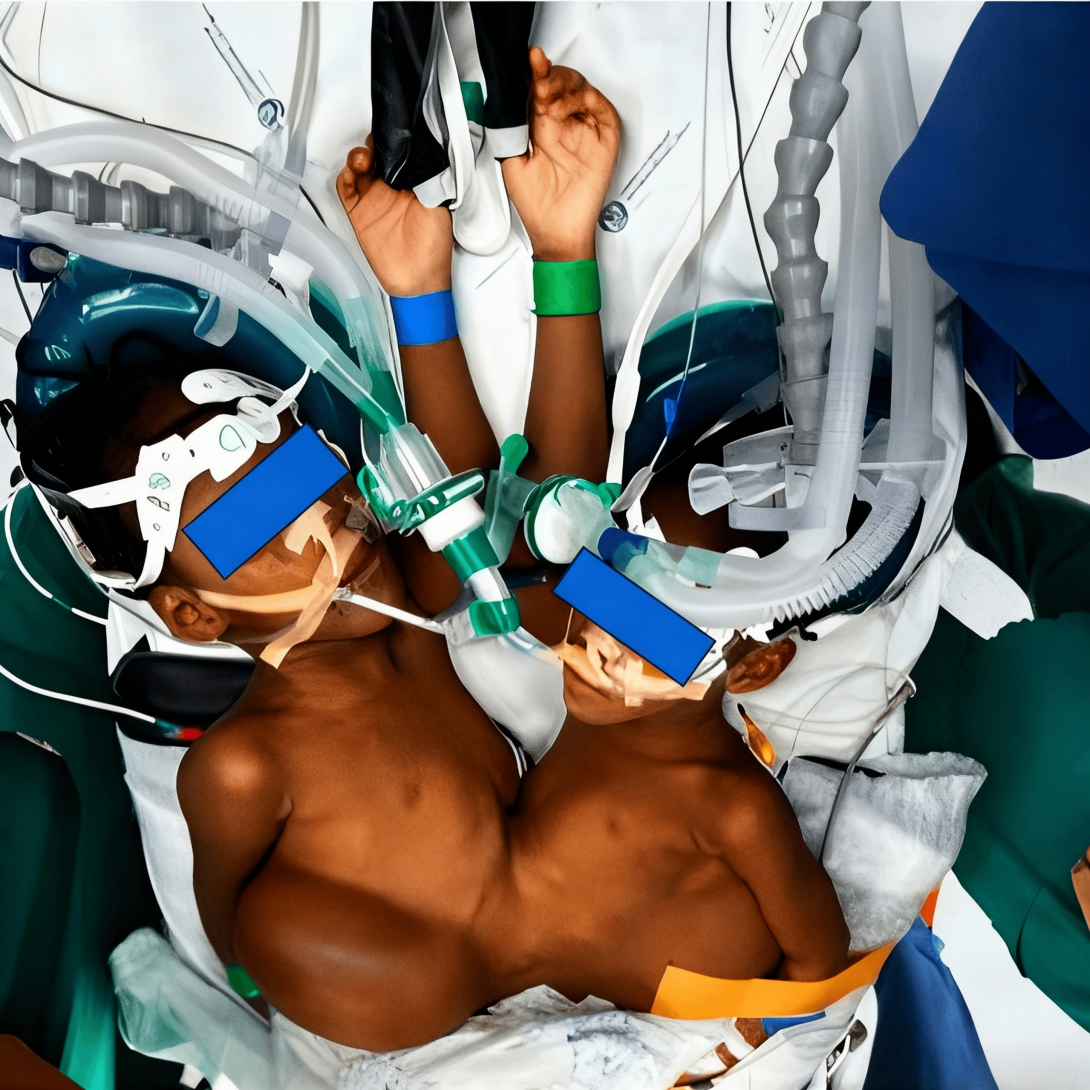
Post-induction positioning of the conjoined twins following sequential anesthetic induction and airway management, highlighting proximity and shared anatomy

Anesthetic induction was performed sequentially using inhalational sevoflurane, followed by maintenance with target-controlled infusion of propofol (Paedfusor TCI model) and remifentanil (0,025 mg/ml perfusion). Airway management was also sequential, with successful endotracheal intubation on the first attempt in both twins (Figure 3) [3,7]. Neuromuscular blockade with cisatracurium (1.5 mg) was administered to the first twin but withheld in the second because of concerns about potential cross-circulation. Mechanical ventilation was maintained using pressure-controlled modes, allowing a coordinated respiratory management of both twins.

Maintenance of anesthesia included meticulous fluid management (20 mL/h) guided and adjusted by hourly balance, invasive hemodynamic monitoring, and proactive blood management strategies. Transfusion therapy was guided by serial laboratory assessment and viscoelastic testing [5,9]. A continuous tranexamic acid infusion (150 mg bolus followed by 5 mg/kg/h perfusion) was initiated before hepatic parenchymal division, and strict normothermia was maintained throughout the prolonged procedure (total time of 18 hours).

Intraoperative course (timeline summary)

Induction and Exposure

Hemodynamic parameters remained stable without the need for vasoactive support.

Hepatic Parenchymal Division

Estimated blood loss was approximately 200 mL. Twin DS received 140 mL of packed red blood cells and 100 mL of plasma. The lowest recorded hemoglobin levels were 7.5 g/dL in Twin DS and 8 g/dL in Twin BS, respectively. Peak intraoperative lactate values were 2 mmol/L and 1 mmol/L, respectively. No sustained hemodynamic instability was observed, and vasoactive support was not required.

Pelvic Osteotomies

These were performed without additional significant blood loss or escalation of vasoactive support.

Following complete separation, each child was transferred to a separate operating room for reconstructive procedures with their previously assigned anesthesia teams. Airway protection, vascular access, continuous monitoring, and color-coded identification were maintained throughout patient transfer and repositioning.

Postoperative course

Both patients were transferred while intubated to the pediatric ICU for continued monitoring, sedation, and ventilatory support. Twin BS was extubated after three days. Mechanical ventilation was maintained for 14 days in twin DS due to postoperative peritonitis caused by Serratia spp. and Pseudomonas aeruginosa, which was treated with targeted antimicrobial therapy and supportive care, resulting in full clinical resolution. The length of stay in the pediatric ICU was 25 days for both patients. Postoperative management focused on hemodynamic stability, electrolyte balance, monitoring hepatic and renal function, multimodal analgesia, and prevention of hypothermia and infection.

Peak postoperative liver enzyme levels were as follows: AST 224 U/L and ALT 260 U/L in Twin DS and AST 218 U/L and ALT 263 U/L in Twin BS, with a progressive downward trend during the intensive care stay. Postoperative transfusion requirements were limited to Twin DS, who received four units of packed red blood cells and one unit of plasma, with a nadir hemoglobin level of 6.2 g/dL. Both patients developed transient pre-renal acute kidney injury, which resolved with supportive management during the pediatric ICU admission. No biliary complications or coagulation disorders were observed in either patient.

## Discussion

The anesthetic management of omphalopagus conjoined twins undergoing elective separation requires meticulous preparation and anticipation of challenges related to shared hepatic tissue and the risk of massive hemorrhage. Consistent with previously published reports, independent anesthesia teams, duplication of equipment, and structured multidisciplinary coordination were central to successful perioperative management in this case [1,6,9]. Shared hepatic parenchyma is widely recognized as a major determinant of anesthetic complexity during omphalopagus twin separation. Previous case reports describe significant intraoperative bleeding during hepatic division, underscoring the importance of invasive monitoring, early activation of blood management strategies, and proactive use of antifibrinolytic agents [2,4,5,10]. In our case, careful planning and controlled surgical progression allowed hemodynamic stability to be maintained throughout hepatic separation.

Sequential anesthetic induction and cautious titration of anesthetic agents are recommended in the presence of potential cross-circulation [3,7]. Although no clinically significant cross-circulation was observed, adherence to these principles minimized the risk of unintended pharmacological effects. Elective separation at an older age offers advantages, including increased physiological reserve, improved tolerance of prolonged anesthesia, and the opportunity for comprehensive preoperative optimization. Our experience supports previous observations that elective timing facilitates safer anesthetic management compared with urgent neonatal separation [3].

Several additional lessons were highlighted in this report. First, while the potential for massive hemorrhage remains a defining concern in omphalopagus separation, this experience demonstrates that anticipatory planning combined with stepwise surgical progression can significantly attenuate physiologic instability. Early definition of transfusion thresholds, pre-induction availability of blood products, and agreement on trigger points for escalation allowed the anesthesia teams to act preemptively rather than reactively. Second, simulation-based multidisciplinary rehearsal proved invaluable in aligning mental models across teams, particularly for high-risk phases such as hepatic transection, patient repositioning, and post-separation transfer to independent operating rooms. These rehearsals enhanced communication efficiency and reduced cognitive load during critical intraoperative transitions.

Advanced multimodal monitoring played a central, decision-guiding role throughout the procedure rather than serving a purely observational function. Continuous invasive arterial pressure monitoring enabled early identification of subtle preload-dependent changes during hepatic division, allowing timely fluid and blood product administration before the development of hypotension. Viscoelastic testing supported targeted, goal-directed transfusion therapy and helped avoid empiric overtransfusion despite prolonged operative duration. Bispectral index monitoring facilitated individualized anesthetic titration, particularly during periods of reduced surgical stimulation and in the context of potential cross-circulation, thereby minimizing the risk of excessive anesthetic depth. Near-infrared spectroscopy provided continuous confirmation of preserved cerebral perfusion during blood loss, prolonged positioning, and the physiological transition following separation, with no episodes of cerebral desaturation observed. Together, these monitoring modalities functioned as an integrated system supporting physiology-based anesthetic management and contributed to the maintenance of hemodynamic and metabolic stability throughout the perioperative course.

This report reinforces that successful anesthetic management of omphalopagus separation does not rely on a single technique, but rather on systematic preparation, adaptability, and adherence to principles informed by cumulative case-based evidence.

## Conclusions

Elective separation of omphalopagus conjoined twins with shared hepatic parenchyma poses substantial anesthetic challenges. Comprehensive multidisciplinary planning, simulation-based preparation, independent anesthesia teams, proactive blood management, and vigilant intraoperative monitoring were essential to achieving a favorable perioperative outcome in our case. This report contributes to the limited body of literature on the topic and reinforces established anesthetic principles for complex conjoined twin separation surgery.
